# Procarcinogens – Determination and Evaluation by Yeast-Based Biosensor Transformed with Plasmids Incorporating *RAD54* Reporter Construct and Cytochrome *P450* Genes

**DOI:** 10.1371/journal.pone.0168721

**Published:** 2016-12-22

**Authors:** Van Ngoc Bui, Thi Thu Huyen Nguyen, Chi Thanh Mai, Yvan Bettarel, Thi Yen Hoang, Thi Thuy Linh Trinh, Nam Hai Truong, Hoang Ha Chu, Vu Thanh Thanh Nguyen, Huu Duc Nguyen, Stefan Wölfl

**Affiliations:** 1 National Key Laboratory of Gene Technology, Institute of Biotechnology (IBT), Vietnam Academy of Science and Technology (VAST), Hanoi, Vietnam; 2 Institute of Pharmacy and Molecular Biotechnology (IPMB), Heidelberg University, Heidelberg, Germany; 3 Thai Nguyen University of Sciences, Thai Nguyen University, Thai Nguyen, Vietnam; 4 Institute of Research for Development (IRD), UMR MARBEC, Montpellier, France; 5 Vietnam National University of Agriculture, Hanoi, Vietnam; Massachusetts Institute of Technology, UNITED STATES

## Abstract

In Vietnam, a great number of toxic substances, including carcinogens and procarcinogens, from industrial and agricultural activities, food production, and healthcare services are daily released into the environment. In the present study, we report the development of novel yeast-based biosensor systems to determine both genotoxic carcinogens and procarcinogens by cotransformation with two plasmids. One plasmid is carrying human *CPR* and *CYP* (*CYP3A4*, *CYP2B6*, or *CYP2D6*) genes, while the other contains the *RAD54-GFP* reporter construct. The three resulting coexpression systems bearing both *CPR-CYP* and *RAD54-GFP* expression cassettes were designated as CYP3A4/CYP2B6/CYP2D6 + RAD54 systems, respectively and used to detect and evaluate the genotoxic potential of carcinogens and procarcinogens by selective activation and induction of both *CPR-CYP* and *RAD54-GFP* expression cassettes in response to DNA damage. Procarcinogens were shown to be predominantly, moderately or not bioactivated by one of the CYP enzymes and thus selectively detected by the specific coexpression system. Aflatoxin B_1_ and benzo(a)pyrene were predominantly detected by the CYP3A4 + RAD54 system, while *N*-nitrosodimethylamine only moderately activated the CYP2B6 + RAD54 reporter system and none of them was identified by the CYP2D6 + RAD54 system. In contrast, the genotoxic carcinogen, methyl methanesulfonate, was detected by all systems.

Our yeast-reporter system can be performed in 384-well microplates to provide efficient genotoxicity testing to identify various carcinogenic compounds and reduce chemical consumption to about 53% as compared with existing 96-well genotoxicity bioassays. In association with a liquid handling robot, this platform enables rapid, cost-effective, and high-throughput screening of numerous analytes in a fully automated and continuous manner without the need for user interaction.

## Introduction

Carcinogens are either genotoxic or nongenotoxic [1]. Genotoxins, such as alkylating agents, can bind to DNA forming DNA adducts and cause damage to the DNA or mutations, which may lead to cancer, while nongenotoxins do not directly cause DNA damage but promote growth or alter the expression or repression of genes by different cellular processes [2, 3]. Conversely, procarcinogens, such as polycyclic aromatic hydrocarbon (PAHs), mycotoxins, etc., become carcinogenic only after they are transformed in metabolic processes including bioactivation by cytochrome P450 monooxygenases (CYPs) [4–6]. These chemicals are found everywhere in the environment, including water, air, soil and food. In Vietnam, a great number of toxic substances, including carcinogens and procarcinogens, e.g. pesticides [7], cadmium, arsenic [8, 9], aflatoxins [10, 11], PAHs [12–14], etc., from industrial and agricultural activities, food production, and healthcare services have been released into the environment in recent years. In 2014, 194 food poisoning outbreaks were reported to the Vietnam Food Administration (VFA), affecting over 5000 people [15]. Thus, development of biosensors for detection of both carcinogens and procarcinogens is of specific interest in Vietnam. Among possible analytical approaches, biosensors offer various advantages over other current analytical methods in particular the possibility of identifying not further specified chemicals. Furthermore, biosensors provide functional information on biological effects, such as cytotoxic and genotoxic effects [16]. In contrast, traditional physicochemical methods, like HPLC or GC-MS, mainly provide analytical information like absolute concentrations of known chemicals [17, 18]. Thus, when substances are new, unknown or not yet deposited in the databases, they cannot be identified by these approaches. Biosensors will not compete but rather complement official physicochemical methods, with specific benefits in environmental monitoring, food safety and quality control, drug testing and other uses where genotoxicity tests are needed to determine potential genotoxic and mutagenic hazards.

Our previous study reported that yeast-based biosensors carrying a green fluorescent protein (GFP)-encoding gene under the control of DNA damage-inducible promoters, *DIN7*, *PLM2*, *RNR2*, or *RAD54*, could be used to identify genotoxic or carcinogenic compounds [19], but were not able to detect procarcinogens, e.g., aflatoxins. This observation can be attributed to the fact that procarcinogenic compounds require biotransformation into carcinogens by cytochrome P450 monooxygenases (CYPs) and NADPH-cytochrome P450 reductase (CPR). Human CYPs belong to the superfamily of membrane-bound proteins that are responsible for the oxidative and reductive metabolism of foreign compounds (xenobiotics) including drugs, steroid hormones, and fatty acids. The detoxification of xenobiotics takes place in two phases. In phase I (functionalisation), the CYPs are responsible for the addition of functional groups to xenobiotics by hydroxylation, dealkylation, deamination, etc.; in phase II (conjugation), transferases use those groups to couple charged molecules making the modified compounds more water soluble so that they can be excreted in the urine [20]. Moreover, for catalytic activity, CYP reactions require the cofactor NADPH as the source of electrons and CPR as the electron transfer partner [21, 22]. In addition to their detoxification function, many CYPs (CYP2B6, CYP2C9, CYP2D6, CYP2E1, CYP3A4, etc.) are also known for bioactivation of harmless chemicals or procarcinogens, e.g. PAHs, nitrosamines, aflatoxins, into their carcinogenic metabolites or carcinogens [23, 24]. It is also documented that many other chemical carcinogens, such as aromatic amines, vinyl and ethyl carbamates, sterigmatocystin, are not active in themselves, however after bioactivation by CYPs the resulting electrophiles can bind covalently to DNA and lead to DNA damage or mutations [25–27]. For example, CYP3A4 enzymatically catalyzes conversion of aflatoxin B_1_ and PAHs, producing their carcinogenic isoforms, CYP2B6 is involved in bioactivation of aflatoxin B_1_ and 4-(methylnitrosamino)-1-(3-pyridyl)-1-butanone (NNK) through hydroxylation reactions, and CYP2E1 contributes to conversion of *N*-nitrosodimethylamine and NNK to mutagenic stereoisomers [20, 26, 27]. Of many such potential carcinogens, the hazardous substances that can arise from untreated industrial wastewater and contaminated food products, such as PAHs, *N*-nitrosodimethylamine, sterigmatocystin, aflatoxin B_1_, have attracted greater public concern in Vietnam. This also led to more regulatory attention. From the beginning of 2015, the Vietnam Food Administration (VFA), Vietnam Ministry of Health warned the population about emerging outbreaks of food poisoning, foodborne and waterborne diseases.

Yeast cells share the same basic cellular components and fundamental biochemical pathways and possess endogenous CYP enzymes for metabolising xenobiotics like mammalian cells. DNA repair mechanisms between yeast and mammalian cells are functionally interchangeable. To determine whether human *CYP* genes could be used to develop yeast-based biosensors for detection of procarcinogens, we improved our previously developed yeast-based biosensor, that could only detect genotoxic carcinogens [19], by transformation with two plasmids: a newly developed plasmid bearing both human *CPR* and *CYP* (*3A4*, *2B6*, or *2D6*) genes, and the already previously used plasmid containing the *RAD54-GFP* reporter construct [16, 19, 28]. In cells transformed with both plasmids and exposed to procarcinogens, CYP enzymes would be responsible for converting a specific substrate into the carcinogenic metabolite able to induce the activity of the DNA damage-inducible *RAD54* promoter triggering expression of GFP. In consequence, the novel yeast-based biosensor presented here would be able to detect both carcinogens and procarcinogens. A set of R packages and Excel macros developed and applied in our earlier studies [19, 29] were used to execute all steps, including liquid handling and pipetting, measurements, data processing and analyzing, of experiments in a fully automated and continuous manner without the need for user interactions.

## Results

Cytochrome P450 monooxygenases (CYPs) have been central to the study of toxicology, since they are involved in metabolism of endogenous molecules, detoxification and biotransformation of xenobiotics, drug-drug and drug-food interactions, and bioactivation of potential carcinogens and other pollutants. Unlike bacterial CYPs, mammalian CYPs require an electron source, the electron transfer partner, such as NADPH-cytochrome P450 reductase (CPR), to show their catalytic activity. Thus, in order to use CYP enzymes as a component of yeast-based biosensors, the activity of CPR and CYPs was first determined.

### Activity of CPR and CYPs

**Fig 1:**
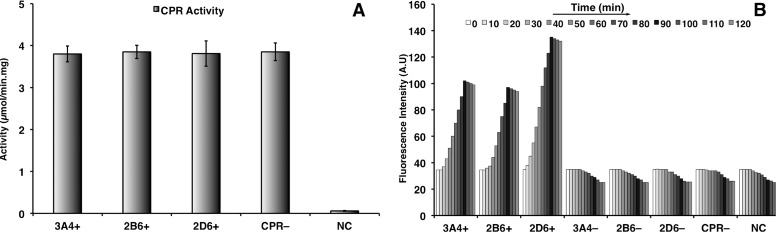
**Activity test of recombinant human CPR (A) and recombinant human CYP3A4, CYP2B6, and CYP2D6 (B)**. 3A4+, 2B6+, and 2D6+ are microsomes of clones coexpressing both *CPR* and *CYP* genes; 3A4–, 2B6–, and 2D6– or CPR–are microsomes of clones expressing only one gene, the *CYPs* or *CPR*, respectively; NC (negative control) are microsomes of clones bearing the control pESC-URA plasmid. The standard deviation values of the measurements of fluorescence microplate assay (B) were all less than 5% of the calculated values and are thus not presented here.

The activity of NADPH-cytochrome P450 reductase (CPR) was detected by reduction of cytochrome c in all microsomes of clones bearing the *CPR* gene regardless of the vector type, either coexpressing both *CPR* and *CYP* genes (3A4+, 2B6+, or 2D6+) or expressing only the *CPR* gene (CPR–). The microsomes of clones transformed with the control pESC-URA plasmid (without heterologous *CPR* gene) did not show any reductase activity (NC; Fig 1A), although *S*. *cerevisiae* possesses its own endogenous oxidoreductase.

For CYP activity, only microsomes containing properly folded CYPs that were confirmed by reduced CO-difference spectra with a peak at 450 nm were introduced to the activity assay. The microsomes of coexpression clones (3A4+, 2B6+, and 2D6+) were probed for activity as they catalyzed the deethylation of the substrate, 7-ethoxycoumarin-3-carbonitrile, to form the fluorescence product, 7-hydroxycoumarin. The formation of this fluorescence product was not observed in single expression clones (3A4–, 2B6–, 2D6–, CPR–, NC; Fig 1B). Since microsomes harboring only one gene *CYP3A4*, *CYP2B6*, or *CYP2D6* (3A4–, 2B6–, 2D6–) or *CPR* (CPR–) did not yield CYP activity, the results confirmed that coexpressed CPR acted as the electron transfer system or redox partner.

Furthermore, the amount of the fluorescence product generated from metabolism of the substrate varied between recombinant CYP enzymes of coexpression clones. The fluorescence signal measured from the 2D6+ clone was apparently higher than that measured from 2B6+ and 3A4+ clones (Fig 1B). The values of kinetic parameters, *Km*, *Vmax* and *Vmax/Km*, determined by nonlinear regression analysis (R function *nls*) for the 3A4+, 2B6+, and 2D6+ clones were 3.5, 3.7, and 2.1 μM (*Km*); 3.2, 2.7, and 4.7 pmol/pmol CYP/min (*Vmax*); 0.9, 0.7, and 2.2 μL/pmol CYP/min (*Vmax/Km*), respectively. The values were independently confirmed by Lineweaver–Burk plot. The 2D6+ clone represented a lower *Km* and a higher *Vmax* value, i.e. higher affinity and higher maximum velocity, as compared with the 3A4+ and 2B6+ clones. These findings not only demonstated the CYP activity of coexpression clones but also showed that CYP2D6 has a higher specificity for this substrate than the other two recombinant CYP enzymes, 2B6+ and 3A4+ (Fig 1B).

### Fluorescence induction of the systems carrying different gene constructs

**Fig 2:**
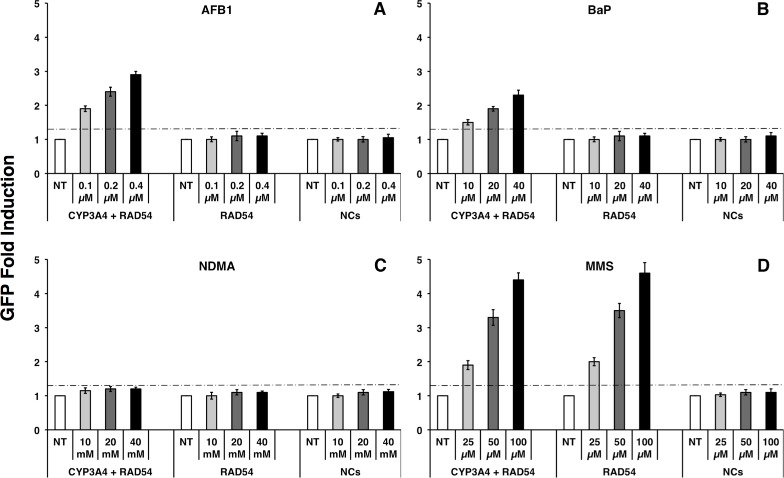
Fluorescence induction in yeast cells transformed with different gene constructs in response to DNA damage. Yeast-based biosensors were either nontreated (NT, control) or exposed to increasing concentrations of AFB1 (A), BaP (B), NDMA (C), and MMS (positive genotoxin, D). CYP3A4 + RAD54: strain transformed with both *CPR-CYP3A4* and *RAD54-GFP* constructs; RAD54: strain transformed with only the *RAD54-GFP* construct; NCs (negative control) system: strain transformed with two control pESC-URA and pUMGP5 plasmids. The GFP fluorescence intensity of measurements was compared within linear range of GFP signals by calculation of GFP fold induction. The horizontal dashed line at 1.3 fold GFP induction is used as cutoff or genotoxicity threshold. Other negative (untransformed yeast cells) and process (medium) controls are not presented here.

In a first effort, the yeast strains bearing different gene constructs were treated with serial dilutions of aflatoxin B_1_ (AFB1), benzo(a)pyrene (BaP), *N*-nitrosodimethylamine (NDMA), and methyl methanesulfonate (MMS) to identify and investigate their genotoxic potential. Fig 2 represents the fluorescence production of three systems: a coexpression system (CYP3A4 + RAD54) harboring both *CPR-CYP3A4* and *RAD54-GFP* constructs in two separate vectors, a single expression systems (RAD54) carrying only the *RAD54-GFP* construct in one vector, and the non-expression system (NCs) bearing two control vectors.

In the present study, the fluorescence intensity of GFP expressed as GFP fold induction [19] was taken from the linear range of the detected signal and should be directly proportional to increasing concentrations of investigated analytes (Fig 2). Similar to the findings from our earlier studies [19, 28], the measured fluorescence signal was dependent on the systems bearing different gene constructs, and on the chemical properties and concentrations of test compounds. The CYP3A4 + RAD54 system produced a signal above the genotoxicity threshold, which was also directly proportional to increasing concentrations of AFB1, BaP, and MMS (Fig 2A, 2B and 2D, respectively) but not to those of NDMA (Fig 2C). For the RAD54 system, the fluorescence signal was only directly proportional to increasing concentrations of MMS (Fig 2D) but not to those of AFB1, BaP, and NDMA, with no significant GFP fold induction below the genotoxicity threshold (≤ 1.3) at all concentrations (Fig 2A, 2B and 2C, respectively). Furthermore, the system harboring only two control vectors (negative control, NCs) produced no signal at any tested concentration of the substances (Fig 2). Thus, the cotransformed CYP3A4 + RAD54 system was able to induce fluorescence when treated with either procarcinogens (AFB1 and BaP) or genotoxic carcinogen (MMS), while the single transformed RAD54 system only produced a fluorescence signal when treated with the genotoxic carcinogen (MMS). The fluorescence induction in response to these investigated compounds was also observed in the other coexpressing systems, cotransformed with vectors including either CYP2B6 or CYP2D6 genes. These alternative coexpression systems contained the following two separate expression vectors, *CPR-CYP2B6* and *RAD54*-*GFP* or *CPR-CYP2D6* and *RAD54-GFP*, respectively.

### Validation and evaluation of fluorescence induction in different coexpressing systems

The three coexpression systems, CYP3A4/CYP2B6/CYP2D6 + RAD54, were exposed to varying serial dilutions of three procarcinogens (AFB1, BaP, NDMA) and a genotoxic carcinogen (MMS, a positive control). The GFP fold induction interpreted as positive (+)/ negative (–) signals [19] of all systems in response to test compounds is summarized in Table 1.

**Table 1:** Analysis and evaluation of fluorescence signals in different yeast strains in response to serial dilution concentrations of test compounds.

Substance	Concentration	CYP3A4 + RAD54	CYP2B6 + RAD54	CYP2D6 + RAD54	RAD54	NCs
Aflatoxin B_1_	NT	–	–	–	–	–
	0.1 μM	+	–	–	–	–
	0.2 μM	++	–	–	–	–
	0.4 μM	++	+	–	–	–
Benzo(a)pyrene	NT	–	–	–	–	–
	10 μM	+	–	–	–	–
	20 μM	+	–	–	–	–
	40 μM	++	–	–	–	–
*N*-nitrosodimethylamine	NT	–	–	–	–	–
	10 mM	–	–	–	–	–
	20 mM	–	–	–	–	–
	40 mM	–	+	–	–	–
Methyl methanesulfonate	NT	–	–	–	–	–
	25 μM	+	+	+	+	–
	50 μM	+++	+++	+++	+++	–
	100 μM	++++	++++	++++	++++	–

CYP3A4 + RAD54; CYP2B6 + RAD54; and CYP2D6 + RAD54: Strains transformed with two *CPR-CYP* and *RAD54-GFP* expression constructs; RAD54: Strain transformed with only one *RAD54-GFP* expression construct; NCs (negative control): Strain transformed with two control pESC-URA and pUMGP5 plasmids. Negative (≤1.3 GFP fold induction),–; positive (>1.3 GFP fold induction), + (1.3, 2]; ++ (2, 3]; +++ (3, 4]; ++++ (4, ∞]

The higher positive signals indicate higher levels of DNA damage whereas negative signals indicate that no genotoxic effect was caused by the compound. Treatment with different concentrations of compounds resulted in varying fluorescence signals or genotoxic results in all systems. The CYP3A4 + RAD54 system exhibited strong positive signals (+, ++) at all treated concentrations of AFB1 and BaP, but showed negative signals (–) when exposed to any concentration of NDMA. The CYP2B6 + RAD54 system produced weak positive signals (+) when treated with even the highest concentrations of AFB1 (0.4μM) and NDMA (40 mM) but negative signals upon treatment with any concentration of BaP. The CYP2D6 + RAD54 system showed negative signals when exposed to any concentration of the three procarcinogens (AFB1, BaP, and NDMA; Table 1), although the 2D6+ system showed a higher affinity and specificity for conversion of 7-ethoxycoumarin-3-carbonitrile as compared with the 3A4+ and 2B6+ (Fig 1). Very strong positive genotoxic signals (+, ++++) were obtained in response to increasing concentrations of MMS with all three systems (Table 1).

Regarding sensitivity and specificity of the systems presented as GFP fold induction (Fig 2) or positive signals (Table 1), the GFP signal obtained was proportional to the concentrations of analytes within a limited linear concentration range, with high concentrations resulting in high GFP signals. A minimum signal but greater than genotoxicity threshold (>1.3 GFP fold induction) was obtained at lower concentrations. Outside the optimal linear concentration range, GFP signals were still detected but no longer in a linear proportional relation of signal intensity to investigated concentrations. The signal tended to decrease when exposed to levels above the highest concentrations of the linear range as the result of cell death. In comparison of the three coexpressing systems, the CYP3A4 + RAD54 system was considerably more sensitive and specific for identifying AFB1 and BaP than the CYP2B6 + RAD54 system, but nonspecific for NDMA. Whereas the CYP2B6 + RAD54 system was shown to be more specific for detecting NDMA but less specific for AFB1 than the CYP3A4 + RAD54 system, and nonspecific for BaP. The CYP2D6 + RAD54 was neither sensitive nor specific for all the three procarcinogens. In respect to genotoxic carcinogen (MMS, a positive control), both coexpressing systems (CYP3A4/CYP2B6/CYP2D6 + RAD54) and single expressing system (RAD54) exhibit a high sensitivity and specificity in determination of MMS, while the system carrying control vectors (NCs) shows neither sensitivity nor specificity for MMS (Table 1). Thus, only the coexpressing systems harboring both *CPR-CYP* and *RAD54-GFP* expression cassettes were able to determine both genotoxic carcinogens and procarcinogens, while systems with a single *RAD54-GFP* construct could detect genotoxic carcinogens only.

### Comparison of test results

In this study, we used a selection of relevant test compounds that had not been investigated before and reevaluated others at different concentrations [19, 30]. The concentrations of test compounds were selected to overlap or the concentration ranges of previously published data sets but also had to include the linear range for detection of the GFP signals in response to test compound treatments (Fig 2, Table 2).

**Table 2:** Summary and comparison of the results of the present study with the data from published report.

Substance	Results from the present study					Results from published report (Walsh et al. 2005)			
	Concentration(μg/mL)	RAD54	CYP3A4 + RAD54	CYP2B6 + RAD54	CYP2D6 + RAD54	Concentration(μg/mL)	RAD54-GFP integrant	RAD54-GFP integrant + CYP3A4	RAD54-GFP integrant + CYP1A2
Aflatoxin B_1_	0.03–0.13	–	+	+	–	20–40	–	Not tested	+
Benzo(a)pyrene	2.52–10.09	–	+	–	–			Not tested	Not tested
*N*-nitroso-dimethylamine	0.74–2.96	–	–*	+	–	1.56–6.25	–	+	Not tested
Methyl methane-sulfonate	2.75–11.01	+	+	+	+	1.02–33	+	+	+

– or +, negative or positive results; –*, varying results inconsistent with those from the original study of Walsh et al. 2005 [30]

The test results determined by the three cotransformants, CYP3A4/CYP2B6/CYP2D6 + RAD54 used in this study, were compared to those analyzed with two other transformants, RAD54-GFP integrant + CYP3A4 or CYP1A2 [30], that were established when the integrated RAD54-GFP strain was transformed with either human *CPR-CYP3A4* or human *CPR-CYP1A2* constructs, respectively. Regardless to sensitivity, all systems were able to detect DNA-damaging substances, with positive results presented as (+), in response to a wide range of concentrations of two procarcinogens, aflatoxin B_1_ and *N*-nitrosodimethylamine, and a positive control, methyl methanesulfonate (Table 2). There are also conflicting data between the two studies, regarding the detection of *N*-nitrosodimethylamine, which was only identified in one CYP3A4 + RAD54 system (–*, +). Moreover, aflatoxin B_1_ and benzo(a)pyrene or benzo(a)pyrene and *N*-nitrosodimethylamine in the referred report were not tested by the RAD54-GFP integrant + CYP3A4 or CYP1A2 systems, respectively (Table 2). The variation in test results between individual systems from two different setups will be discussed later.

## Discussion

According to the statistics report of the Vietnam Food Administration (VFA), 250–500 outbreaks of foodborne and waterborne diseases caused by hazardous substances are annually reported since 2008–2014. Thus, development of bioanalytical tools to quickly identify hazardous substances including genotoxic carcinogens and procarcinogens is of great current interest in Vietnam. However, many organic exogenous chemical carcinogens are procarcinogens that are harmless substances and do not cause cancer by themselves, but need to be bioactivated by cytochrome P450 enzymes (CYPs) before forming DNA or protein adducts. Furthermore, the stable and bulky DNA adducts cannot be simply repaired by the different repair systems [31]. It was documented that CYP2C8, -2C9, and -2D6 enzymes contribute less to bioactivation of procarcinogens into electrophiles or ultimate carcinogens than CYP1A1, -1B1, -2A6, -2A13, -2E1, and -3A4 enzymes [26]. Thanks to the remarkable gene homology between yeast and human cells, yeast cells provide an excellent cell model for toxicity assays. About 40% of yeast genes share conserved amino acid sequences with known or predicted human proteins [32]. Furthermore, fundamental biochemical pathways and cellular processes are conserved between yeast and humans, and about 30% of human genes known to be involved in human diseases have orthologs in yeast [33]. Thus, genetically modified yeast cells provide an excellent model for the 3R concept (reduction, replacement, and refinement) in toxicology and ecotoxicology, and are ideal as cell based bioanalytical, biosensor tools due to the simple cultivation and lack of ethical problems. Although, yeast has at least three functional endogenous CYP type enzymes, which can bind various chemical carcinogens [34, 35], the yeast CYP enzymes have other substrate specificities in comparison to the human CYP enzymes.

Therefore, the aim of this study was to use recombinant human CYP enzymes to develop a yeast-based biosensor for the detection of both genotoxic carcinogens and procarcinogens that can be widely used for the evaluation of genotoxic risk potential. We used these biosensors to identify and assess four chemical compounds, a genotoxin (MMS) and three procarcinogens (AFB1, BaP, and NDMA) that were not investigated or detected in the range of concentrations used here by previously developed systems [16, 19].

The fluorogenic substrate, 7-ethoxycoumarin-3-carbonitrile, has been used to determine the activity of CYP1A family enzymes as it is converted to a fluorescent product, 7-hydroxycoumarin. In our experiments it was suitable for continuous determination of cytochrome P450 mixed-function monooxygenases, such as CYP3A4, CYP2C9, CYP2B6, CYP2D6, since CYPs have broad and overlapping substrate specificities [36, 37]. Single expression of human CYP3A4, CYP2B6, and CYP2D6 did not yield any CYP activity, while coexpression together with human NADPH-cytochrome P450 reductase (CPR) resulted in clearly measurable activity expressed as amount of fluorescent product formed per minute (Fig 1B). The present results support the notion that CYP reactions require the cofactor NADPH as the source of electrons and CPR as the electron transfer partner [22]. These findings also indicate that the endogenous CPR of *S*. *cerevisiae* is incompatible with human CYP3A4, CYP2B6, and CYP2D6. A similar incompatibility had been observed in *Pichia pastoris* [21]. Regarding the enzymatic efficiency of coexpressing clones measured by the fluorogenic substrate conversion, the 2D6+ clone exhibited a higher catalytic activity in biotransformation of the substrate into the fluorescence product than the 3A4+ and 2B6+ clones (Fig 1B). The higher affinity and substrate conversion rate of 2D6+ clone was also confirmed by the lower *Km* and higher *Vmax* values as compared with those of 3A4+ and 2B6+ clones (*Km* ~ 2.1, 3.5, and 3.7 μM and *Vmax* ~ 4.7, 3.2, and 2.7 pmol/pmol CYP/min for the 2D6+, 3A4+, and 2B6+ clones, respectively). In addition, some studies reported that 7-ethoxycoumarin-3-carbonitrile together with dextrometorphan were two typical substrates for CYP1A2 and CYP2D6 [21, 37].

When individual cotransformants harboring both *CPR-CYP* and *RAD54-GFP* expression cassettes in two separate vectors to form the three reporter strains designated as the CYP3A4 + RAD54, CYP2B6 + RAD54, and CYP2D6 + RAD54 systems, these systems produced distinct fluorescence or positive signals in the presence of different concentrations of AFB1, BaP, and NDMA (Fig 2 and Table 1). These individual responses may be explained by the fact that CYPs probably converted procarcinogens, AFB1, BaP, and NDMA, into several metabolic products including genotoxic and non-genotoxic metabolites. Of which only genotoxic metabolites were able to induce the activity of the DNA-damage inducible *RAD54* promoter leading to expression of *GFP*, while non-genotoxic metabolites were not. For example, CYP3A4, an enzyme mainly expressed in the liver, is known to oxidize AFB1 into several subproducts, AFB1-exo-8,9-epoxide, AFB1-8,9-endo-epoxide, and AFB1-3 known to [6, 38]. But only the AFB1-exo-8,9-epoxide stereoisomer is a mutagenic metabolite, which reacts efficiently with DNA at the N7 position of guanine to form AFB1-N7-Gua adduct and induce G-to-T transversions [38–40]. Thus, AFB1-exo-8,9-epoxide was capable of activating the *RAD54* promoter to drive *GFP* expression producing fluorescence signals (Fig 2A and Table 1). CYP3A4 is also involved in BaP transformation [23]. CYP3A4 presumably metabolized BaP into BaP-3-hydroxy, BaP-9-hydroxy, BaP-4,5-dihydrodiol, BaP-7,8-dihydrodiol, and the ultimate genotoxic metabolite, BaP-7,8-dihydrodiol-9,10-epoxide (diol epoxide). The reaction mechanism is similar to that of AFB1, in which this diol epoxide covalently binds to DNA at the N7 position of guanine [41, 42], thereby inducing *RAD54* promoter and downstream *GFP* expression (Fig 2B and Table 1).

Like CYP3A4, CYP2B6 is also capable of biotransformation of AFB1 to a potent mutagen producing positive signals but much less than those produced by the CYP3A4 strain (Table 1). In fact, some studies reported that human CYP2B6 was responsible for metabolism of AFB1 to carcinogenic derivatives [43, 44]. The major activation pathway of AFB1 by CYP2B6 and CYP3A4 to form an active mutagen, AFB1-exo-8,9-epoxide, could be the same [44, 45]. CYP2B6 also had some activity in metabolic activation of a nitrosamine compound, NDMA, which was not activated by CYP3A4 (Table 1). The CYP2B6-mediated conversion of NDMA probably led to form an alkyl-diazonium ion causing the carcinogenic effect through covalent binding to DNA [26, 46]. Thus, these CYP2B6-mediated covalent DNA adducts were able to trigger the *RAD54*-*GFP* expression cassette generating low positive signals (Table 1).

In contrast to CYP3A4 and CYP2B6, CYP2D6 seems not to be involved in the enzymatic activation of the three procarcinogens to their respective genotoxic metabolites, or biotransformation by CYP2D6 only resulted in nongenotoxic metabolites unable to activate the *RAD54* promoter. Some studies reported that CYP2D6 is only weakly or not involved in bioactivation of procarcinogens including 4-(methylnitro-samino)-1-(3-pyridyl)-1-butanone (NNK) or AFB1, BaP, and NDMA to their active carcinogenic stereoisomers [27, 47, 48].

It must be kept in mind that the yeast cell wall, the outer membrane, and associated proteins, including ATP-binding cassette (ABC) transporter proteins present a potential barrier to influx and efflux, or promote active efflux of a wide range of drugs and chemical compounds. According to Lipinski’s rule of five (RO5) a drug-like compound typically has a molecular mass less than 500 [49], which fits well to the compounds in this study that are all less than 500 g/mol. To improve sensitivity in connection with import and export mechanisms, Walsh et al. (2005) [30] established a collection of yeast strains in which single or multiple genes (*pdr5*, *erg6*, *snq2*, *yor1*) required for cell wall integrity and/or multi-drug resistance were deleted. In general, a single cell wall mutant was not effective in improving the detection of genotoxins, while double or multiple cell wall mutants showed more sensitivity to growth inhibition than genotoxicity when exposed to such genotoxins compared with the wild-type strain [30].

Thus, the varied specificity and sensitivity of the coexpressing systems for detection of procarcinogens could either be due to different efficiencies in enzymatic processing by the CYPs or due to varying ratios of genotoxic to non-genotoxic products. Indeed, mycotoxins, such as AFB1, and PAHs, such as BaP, were known to be predominately and moderately catalyzed by human CYP3A4, respectively, while *N*-nitrosamines, such as NDMA, were moderately catalyzed by CYP2B6. Moreover, CYP3A subfamily enzymes favor or prefer formation of AFB1-exo-8,9-epoxide to AFB1-3 alpha-hydroxy metabolite as compared with CYP2B subfamily enzymes [20, 27, 50]. Taken together, DNA damage, *RAD54* promoter activity, and consequently positive signals would be induced at different levels. Nevertheless, MMS, a genotoxic carcinogen that directly modifies DNA both *in vitro* and *in vivo*, without metabolic activation by methylation on N7-deoxyguanosine and N3-deoxyadenosine to form base mispairing and replication blocks [51, 52], caused a stronger DNA damage effect and more consistent positive signals in all systems (Table 1). Our earlier reports also show that a broad range of genotoxins was able to directly modify DNA and induced the *RAD54-GFP* expression construct, subsequently resulting in strong positive signals [19, 28].

In comparison with previously published data (Table 2) indicated as a single negative (–) or positive result (+), there is agreement that only the strains harboring both *CPR-CYP* and *RAD54-GFP* constructs were capable of identifying two procarcinogens, aflatoxin B_1_ and *N*-nitrosodimethylamine, while the systems carrying only the *RAD54-GFP* construct were not (Table 2). In case of conflicting data (–* and +) concerning the use of the CYP3A4 + RAD54 system (in this study) and RAD54 integrant + CYP3A4 system [30] in detecting *N*-nitrosodimethylamine, which was deduced as negative result (–*) in this study, but positive (+) in the published report. This inconsistency could be due to different experimental protocols, setups or designs. For example, the negative result for *N*-nitrosodimethylamine in the CYP3A4 + RAD54 system in this study could be due to low concentrations of this substance used. In consequence, the tiny amounts of its metabolic products were not able to induce the RAD54 promoter-driven GFP expression in the coexpression system. However, the same low concentration of this compound was detected by the CYP2B6 + RAD54 system in this study, presumably due to better conversion into the genotoxic metabolite with this CYP isoenzyme. It should be noted that the concentrations used in this report were generally lower than in earlier studies. The CYP3A4 + RAD54 system in this study produced a positive result when exposed to low concentrations (0.03–0.13 μg/mL) of aflatoxin B_1_, while the RAD54 integrant + CYP1A2 system in the published report generated the positive result in response to higher concentrations (20–40 μg/mL) of this substance (Table 2). This could be explained by predominant bioactivation of aflatoxin B_1_ by the CYP3A4 enzyme. Taken together, these findings support the view that no single test is sufficient to adequately identify and evaluate all toxic compounds or drug candidates and their potential mutagenic or carcinogenic hazards and risks to animals and humans.

Our novel yeast-reporter genotoxicity assays performed in 384-well microplates (70 μL total volume per well) instead of using four 96-well microplates (150 μL total volume per well) allow to reduce the total volume of required chemicals by 53% when compared to the 96-well format used in the previous studies [16, 28, 30]. In association with a computer-controlled automated laboratory system developed and used in our previous studies [19, 29], this incorporation facilitates rapid, cost-effective, and high-throughput screening of both genotoxic carcinogens and, in particular, procarcinogens, AFB1, BaP and NDMA, that were not detected by our previous systems [19] and GreenScreen [16]. On the other hand, these systems probably lend themselves to evaluate other compounds with similar properties or new substances. For example, the CYP3A4 + RAD54 system could be used to test new mycotoxin, sterigmatocystin, or other mycotoxin, aflatoxin G_1_ (AFG1), or other PAH, benzo[c]phenanthrene (BcP), because CYP3A4 enzymatically bioactivated AFG1 or BcP into AFG1-8,9-epoxide or diol epoxide stereoisomers that were able to intercalate into the DNA helix or covalently bind to DNA, respectively [23, 38, 53]. The CYP2B6 + RAD54 system could be used to determine other procarcinogens, since CYP2B6 can contribute to a broad range of procarcinogen activation reactions [43]. Of which were *N*-nitrosamines, 4-(methylnitrosamino)-1-(3-pyridyl)-1-butanone (NNK), as NNK was metabolically activated through CYP2B6-catalyzed alpha-hydroxylation to produce several genotoxic metabolites, two of which were 4-(3-pyridyl)-4-oxo-butyl (diazohydroxide) and methane diazohydroxide covalently binding to DNA and methylating DNA to form DNA adducts, respectively [54].

Thus, the yeast-based biosensors presented in this study provide a clear advantage to our previous systems. The newly developed systems can be used as a single systems for the detection of both carcinogens and procarcinogens, while the earlier systems could identify only genotoxic carcinogens [19, 28]. It also should be noted that all comparable systems detect carcinogenic compounds only when a certain threshold concentration sufficient for triggering DNA-damage in the yeast strains is reached. Below this threshold concentration no signal will be detected. Moreover, these systems are particularly appropriate for evaluating immediate genotoxic damage, while delayed genotoxic damage triggered by low levels of contaminations, which may lead to DNA damage after extended exposure, is not accessible with these systems.

The present systems contribute to the development of yeast-based biosensors for cytotoxicity and genotoxicity, and are geared toward screening rather than analytical test. Also, they will supplement existing analytical tools and could be applied in environmental monitoring and food quality control. Indeed, mycotoxins and PAHs released from agricultural and industrial activities are considered to be a major public health problem in developing countries like Vietnam where long-term food storage is often inadequate for high heat and humidity, that typically induces the growth of mold, and untreated or inadequately treated wastewater is discharged into the environment.

Based on the findings presented here further improvements could be performed including the use of alternative promoters of DNA damage-inducible genes of *S*. *cerevisiae*, such as *RNR2 PLM2* or *DIN7* [19, 28], or other human *CYP450* genes. For example, CYP1A1 and CYP2E1 play a major role in bioactivation of PAHs and *N*-nitrosamines into mutagenic metabolites, respectively [27, 55]. Apart from that, the sensitivity of yeast-based genotoxicity assays can be further enhanced by modification of the cell wall permeability in mutant yeast strains [30], and pretreatments increasing the biological activity of compounds in yeast cells. In general, the yeast-based biosensor systems will be useful in any application where genotoxicity assays are required to assess the genotoxic hazards as in drug discovery for early testing of drug candidates.

The coexpressing systems presented here that harbor both *CPR-CYP* (*CPR-CYP3A4*, *-CYP2B6*, or *-CYP2D6*) and *RAD54-GFP* expression cassettes, were able to determine genotoxic carcinogens as well as procarcinogens. In contrast, reporter systems with the *RAD54-GFP* construct alone could detect only genotoxic carcinogens. Performance of these genotoxicity assays in 384-well microplates will allow to reduce chemical consumption to about 53% as compared with existing 96-well genotoxicity bioassays [16, 19, 28, 30]. In association with a computer-controlled automated laboratory system, this liquid handling platform enables rapid, cost-effective, and high-throughput screening of numerous analytes in a fully automated and continuous manner without the need for user interactions.

## Materials and methods

### Yeast strains, plasmids, chemicals

The *Saccharomyces cerevisiae* strain Y486 (also known as FF 18984, *MATa leu2- 3*,*112 ura3-52*, *lys2-1*, *his7-1)*, the reporter plasmid, *RAD54-GFP* pUMGP5, and vectors harboring genes that encode a human NADPH-cytochrome P450 reductase (CPR; EC 1.6.2.4) and three human cytochrome P450 monooxygenases (CYPs; EC 1.14.14.1 and EC 1.14.13.x), CYP3A4, CYP2B6, and CYP2D6, were provided by Stefan Wölfl. The pESC plasmid containing a yeast URA3 selection marker (pESC-URA, pESC Yeast Epitope Tagging Vectors, Cat. # 217454) used for expression of *CYP* and *CPR* genes was purchased from Stratagene (Agilent Technologies, Stratagene, Santa Clara, CA, USA).

All test compounds used for our genotoxicity assays were purchased from Sigma-Aldrich (Taufkirchen, Germany): aflatoxin B_1_ (AFB1, CAS No. 1162-65-8), benzo(a)pyrene (BaP, CAS No. 50-32-8), *N*-nitrosodimethylamine (NDMA, CAS No. 62-75-9), and one positive genotoxicity control, methyl methanesulfonate (MMS, CAS No. 66-27-3). Other chemicals, reagents or substances used in this study were purchased from Merck (Darmstadt, Germany) and Fisher Scientific (Germany).

### Construction of pESC-URA plasmids containing *CPR* and *CYP* genes

The primer pairs listed in Table 3 were used to amplify *CPR*, *CYP3A4*, *CYP2B6*, and *CYP2D6* genes by PCR. The PCR procedure was performed as described previously on a Mastercycler pro (Eppendorf, Hamburg, Germany) [19]. The PCR products were examined by 1% agarose gel electrophoresis analysis (Bio-Rad, Munich, Germany) and extraction and purification (QIAquick Gel Extraction Kit, Qiagen, Hilden, Germany).

**Table 3:** Primer pairs used for construction of plasmids pESC-*CPR-CYPs*

Primer name	Sequence (5′-3′)	Size of PCR product (bp)
CPR-F	GCC**GGATCC**ATGGGAGACTCCCACGTGGA	2064
CPR-R	GG**GGTACC**CTAGCTCCACACGACCAGGG	
CYP3A4-F	GC**CACTAGT**ATGGACCTCATCCCAAATTT	1539
CYP3A4-R	GG**TTAATTAA**TCATTCTCCACTTAGGGTTC	
CYP2B6-F	GCC**ACTAGT**ATGGAACTCAGCGTCCTCCT	1506
CYP2B6-R	GG**TTAATTAA**TCAGCGGGGCAGGAAGCGGAT	
CYP2D6-F	GCC**ACTAGT**ATGGGGCTAGAAGCACTGGT	1524
CYP2D6-R	GG**TTAATTAA**CTAGCGGGGC ACAGCACAAA	

The underlined and bold bases are the restriction sites of *Bam*HI (GGATCC) and *Kpn*I (GGTACC); *Spe*I (ACTAGT) and *Pac*I (TTAATTAA) incorporated in forward (F) and reverse (R) primers for amplification of *CPR* and *CYPs* genes (*CYP3A4*, *CYP2B6*, and *CYP2D6*), respectively. The extra bases upstream of the restriction sites are for improvement of cutting efficiency.

Firstly, the purified PCR product, *CPR* fragment (≈ 2064 bp), and pESC-URA plasmid were digested with each enzyme, *Bam*HI and *Kpn*I, (New England Biolabs, NEB, Frankfurt, Germany) in a separate reaction and purified (QIAquick Spin PCR Purification Kit, Qiagen). Prior to ligation reaction, the nicked pESC-URA plasmid was dephosphorylated by antarctic phosphatase (NEB) for preventing recircularisation. The *CPR* fragment was then joined by ligation (T4 DNA ligase, NEB) into digested pESC-URA plasmid to form pESC-URA plasmid containing *CPR* insert, hence called pESC-*CPR*. These enzymes were all used and inactivated (if necessary) according to the instruction of the manufacturer (NEB). The newly formed plasmid was transformed in homemade chemically *E*. *coli* competent cells (DH5α™; Invitrogen, Darmstadt, Germany) by the conventional KCM (KCl, CaCl_2_, and MgCl_2_) transformation method. The transformants were selected by plating on LB agar (Miller's LB broth base, Invitrogen) supplemented with ampicillin (100 μg/mL). The ligation product, pESC-*CPR* plasmid, was then purified (QIAprep Spin Miniprep Kit, Qiagen), digested with *Bam*HI and *Kpn*I, and the digests of ligation were checked by separation in agarose gel same as mentioned above.

Next, the other three purified PCR products, *CYP3A4*, *CYP2B6*, *CYP2D6* fragments, with the expected size (Table 3) and the pESC-*CPR* plasmid were digested with each enzyme, *Spe*I and *Pac*I (NEB), respectively. The same procedure and steps were performed as described above to obtain three newly formed plasmids, pESC-*CPR*-*CYP3A4*, *-CYP2B6*, and *-CYP2D6*. Subsequently, the concentration of the purified plasmids was determined (NanoDrop 2000, Thermo Scientific, Dreieich, Germany) and the sequencing primers provided in the kit (pESC Yeast Epitope Tagging Vectors) were used to sequence the *CYPs* and *CPR* genes (ABI Prism® 3100 Genetic Analyzer, Applied Biosystems, USA).

### Determination of enzymatic activity of CPR and CYPs

The individual plasmids were transformed into wild type *S*. *cerevisiae* cells (strain Y486) using the LiAc/SS carrier DNA/PEG method developed by Gietz and Woods [56]. The transformants and recombinant plasmids were maintained during cell growth and division by further selection for uracil prototrophy in SD/-Ura agar (Clontech, TaKaRa, France). The recombinant proteins, CPR and CYPs, were expessed when the transformants were cultured in SD/-Ura medium containing 2% galactose and 0.5% raffinose at 30°C with shaking. After 24 hours of cultivation in main culture, yeast cells were harvested by centrifugation (3000 g, 4°C, 10 min). Pellets were resuspended in homogenisation buffer (50 mM potassium phosphate, pH 7.9; 1 mM EDTA; 5% Glycerol; 2 mM DTT; 1 mM PMSF) to 20 OD_600_ units of yeast cells. Cell suspension was added with 1 g acid-washed glass beads (0.4–0.5 mm in diameter, Sigma Aldrich). Cell disruption was performed by vortexing (3x5 min with cooling on ice in between) in Mixer Mill MM 300 (Retsch, Haan, Germany). The supernatant was separated from cell debris and glass beads by centrifugation at 14000 g, 4°C for 15 min (Hettich, Tübingen, Germany). Then, the supernatant was ultracentrifuged at 100000 g and 4°C for 1 h (Beckman Coulter, Krefeld, Germany), the microsomal pellet obtained (CPR or CYP microsomal protein) was resuspended in homogenisation buffer and used immediately for enzymatic assays. The protein concentration was determined using the method of Bradford (1976). The CYP concentration was determined by reduced carbon monoxide (CO) spectra that was measured by the method according to Omura [57]: 100 μg microsome protein in sodium phosphate buffer (0.1 M, pH 7.4) containing 10% glycerol and 0.5% Triton X 100 were incubated on ice for 10 min. 3 to 5 mg N_2_S_2_O_4_ were added and the solution was then transferred into UV-cuvettes and a reference spectrum was recorded from 400 to 500 nm by SmartSpec Plus UV/Vis Spectrophotometer (Bio-Rad, Munich, Germany). The reaction was started by aerating with CO gas for 30 seconds and the spectrum was remeasured. The CYP concentration was calculated using Beer Lambert law and demonstrated by following equation: [CYP] (μM) = ΔOD_450-490 nm_.f/ε.d, where ΔOD_450-490 nm_ is the absorbance difference at 450 and 490 nm, f is the dilution factor, ε is the extinction coefficient (91 mM^-1^ cm^-1^), and d is the cuvette thickness (1 cm).

#### CPR activity assay

The determination of CPR (NADPH-cytochrome P450 reductase) activity was performed essentially as previously described [58, 59]: The CPR activity was spectrophotometrically measured by the rate of reduction of cytochrome c in the presence of NADPH (Sigma Aldrich). 500 μg cytochrome c (Sigma Aldrich) in potassium phosphate buffer (50 mM, pH 7.5) were mixed with 100 μg microsomal protein and filled up with potassium phosphate buffer to 950 μL. The reaction was started by adding 50 μL of fresh aqueous NADPH solution (12 mM). The absorbance change was recorded at 550 nm for 20 seconds using SmartSpec Plus UV/Vis Spectrophotometer (Bio-Rad). The CPR activity was calculated using equation based on Beer Lambert law: ΔOD_550/min_/ε, where OD_550_ is the absorbance change measured at 550 nm, ε is extinction coefficient of 21 mM^-1^ cm^-1^. One enzyme unit is defined as μmol/min.

#### CYP activity assay

The activity of CYPs was monitored by fluorescence-based assay according to Donato and Favreau with modifications [37, 60]: These activity assays were based on the deethylation of 7-ethoxycoumarin by CYPs to the fluorescence product, 7-hydroxycoumarin. A substrate solution comprising 2.5 mM 7-ethoxycoumarin-3-Carbonitrile (Sigma Aldrich) in potassium phosphate buffer (100 mM, pH 7.4), 3 mM NADPH, and 0.02% (v/v) Pluronic F-68 (Sigma Aldrich) was pre-incubated in a 96-well microplate with black walls and transparent flat bottoms (8x12 well format, Greiner Bio-one, Germany) at 37°C for 5 min and then mixed with 200 μg CYP microsomal protein in potassium phosphate buffer (100 mM, pH 7.4) to reach a total reaction volume of 250 μL per well. The fluorescence signal was measured at 405 nm (excitation wavelength) and 460 nm (emission wavelength) and recorded every 10 min for a total of 120 min using microplate reader (Safire^2^ Microplate Reader, Tecan, Switzerland) controlled by XFLUOR4 SAFIRE II software (Xfluor Excel macros, Version: V 4.62n for Safire^2^ Microplate Reader). In addition, the kinetic parameters (*Vmax*, *Km*) were determined from enzyme activities at 10 different substrate concentrations (1–100 μM) by either Lineweaver–Burk plot or performing a direct nonlinear regression of the Michaelis Menten model. The *Vmax* and *Km* constants of this model were determined by minimizing the sum of squared differences between predicted model and measured data using the R function *nls* that was described in our previous study [29].

### Development of novel yeast-based biosensor

Yeast cells (strain Y486) were co-transformed with two different kinds of plasmids. One that was formed in this study and described above beared both the *CPR* and *CYP* genes (*CPR-CYP3A4*, *CPR-CYP2B6*, or *CPR-CYP2D6* construct); the other one that was created and used in previous studies carried *RAD54-GFP* reporter construct [16, 19, 28]. For the cotransformation, the protocol was the same as mentioned above, but the SD/-Ura medium was supplemented with 0.2 mg/mL of geneticin antibiotic (G418) for the selection and maintenance of cotransformants. Since the discriminative cotransformants carried only one of *CYP* genes different from another (*CYP3A4*, *CYP2B6*, or *CYP2D6*), they were hence designated as CYP3A4/CYP2B6/CYP2D6 + RAD54 strain or system, respectively.

Such cotransformants, called novel yeast-based biosensors, were used to investigate the potential genotoxicity of chemical compounds by genotoxicity assay. Four test compounds were selected and prepared in stock solutions as follows: 0.8 μM aflatoxin B_1_ (249.82 ng/mL; AFB1), 80 μM benzo(a)pyrene (20.18 μg/mL; BaP), 80 mM *N*-nitrosodimethylamine (5.92 mg/mL; NDMA), and one positive genotoxicity control, 0.1 mM methyl methanesulfonate (11.01 μg/mL; MMS) were diluted in F1-Ura medium plus 4% DMSO (v/v). All components of F1-Ura medium, except that glucose was substituted with an equivalent concentration of galactose plus 0.5% raffinose and 0.02% G418, were the same as previously described [19]. Also, the performance of genotoxicity assay including the steps: experimental design and setup; fluorescence measurement; data acquisition and analysis; threshold calculation; and plotting were adequately described in our previous study [19].

In brief, the assay was performed in 384-well microplate (24 x 16 well format; Greiner Bio-one, Germany) with black walls and transparent flat bottoms. Each well was pipetted in triplicate for determining mean values, standard deviation (SD), and the assay was repeated at least 3 times for reproducibility. Except for the preparation of stock compound solutions and sealing the microplate with breathable membrane (Diversified Biotech, USA), all these steps were automatically executed by a combination of robotic system (Genesis RSP-150 Liquid Handling System, Tecan, Switzerland) and microplate reader (Tecan). A set of R-scripts developed in our previous study [29] controls both the robot and reader by translating the instructions into machine commands, executing liquid handling and measurements, collecting the data, and processing it without user interaction. In the present study, all the fluorescence measurements were carried out after 16 h of incubation at 30°C with agitation in this reader. The data were saved either in file.RData or exported to Microsoft Excel for conventional data processing and analysis, and plotting (bar graphs with SD values) by the Excel macros.
